# Flat teams drive scientific innovation

**DOI:** 10.1073/pnas.2200927119

**Published:** 2022-06-03

**Authors:** Fengli Xu, Lingfei Wu, James Evans

**Affiliations:** ^a^Knowledge Lab, University of Chicago, Chicago, IL 60637;; ^b^Department of Sociology, University of Chicago, Chicago, IL 60637;; ^c^School of Computing and Information, University of Pittsburgh, Pittsburgh, PA 15260

**Keywords:** science of science, teams, hierarchy, networks, group structure

## Abstract

With teams growing in all areas of scientific and scholarly research, we explore the relationship between team structure and the character of knowledge they produce. Drawing on 89,575 self-reports of team member research activity underlying scientific publications, we show how individual activities cohere into broad roles of 1) leadership through the direction and presentation of research and 2) support through data collection, analysis, and discussion. The hidden hierarchy of a scientific team is characterized by its lead (or L) ratio of members playing leadership roles to total team size. The L ratio is validated through correlation with imputed contributions to the specific paper and to science as a whole, which we use to effectively extrapolate the L ratio for 16,397,750 papers where roles are not explicit. We find that, relative to flat, egalitarian teams, tall, hierarchical teams produce less novelty and more often develop existing ideas, increase productivity for those on top and decrease it for those beneath, and increase short-term citations but decrease long-term influence. These effects hold within person—the same person on the same-sized team produces science much more likely to disruptively innovate if they work on a flat, high-L-ratio team. These results suggest the critical role flat teams play for sustainable scientific advance and the training and advancement of scientists.

Teams are the engines of modern science, having grown in prevalence and size across all areas of scientific and scholarly investigation (1). Despite the known importance of team structure in many domains of economy and society, little is known about how team structure in science relates to innovation and discovery outcomes, from a lack of consistent, large-scale data. Previous experimental and observational studies of emergent team structure reveal that flatter teams with more balanced (2) or synchronous communication between members (3, 4) achieve higher performance in problem-solving (2), sales (3), trading (4), and healthcare (5) settings, partially because coordinated attention facilitates the adaptability needed to respond to uncertainty, complexity, and change (3). Hierarchy, by contrast, accelerates rapid top-down communication and efficiency (6), but necessarily reduces symmetric coordination (7) and yields greater inequality in team member benefits, ranging from higher deaths in mountaineering expeditions (6) to uneven sacrifices in markets (8).

Calls for more transparent, honest, and equitable credit from the open science movement have inspired increased mandatory reporting for individual research contribution on published papers in most high-profile journals. Reporting has become increasingly standardized to more accurately reflect researcher contribution and signal contributor skills. In this paper, we use contributor-level information to explore the relationship between the hierarchy of individual team contributions and the character of the team’s contribution to unfolding scientific advance. Recent studies analyzed the division of labor across stated scientific contributions (9, 10), but did not explore the hierarchical research roles that emerge from the inequality of contributions [e.g., “lead” versus “supporting” team members (11)].

Here, we demonstrate how specific scientist contributions cohere into hierarchical roles that lead or follow in support of research publication and yield a simple lead (or L) ratio associated with each paper of *n* authors ranging from 1/n for maximum hierarchy to 1.0 for flatness. Teams with higher L ratios broadly share leadership opportunities in fulsome collaboration, while those with low L ratios segregate leading from supporting contributions. We validate these patterns with the position of authors in paper bylines, the imputed ideas and prior knowledge each scientist contributes to each paper, and the history of contributions scientists have made to science as a whole. These signals are available for all papers and enable robust extrapolation from papers with self-reports to all papers in science. These patterns reveal how team hierarchy may emerge as teams grow. When it does, contributions for science differ dramatically.

Teams with higher L ratios manifest greater novelty in their atypical combination of ideas (12), while those with lower L ratios engage in development of established research directions (13). Teams with higher L ratios facilitate greater productivity for the average author, while those with low L ratios amplify the productivity for just those on top. Finally, teams with higher L ratios are associated with the potential for long-term scientific influence, while those with lower L ratios contribute to ensured short-term attention.

## Results

**Fig. 1.**
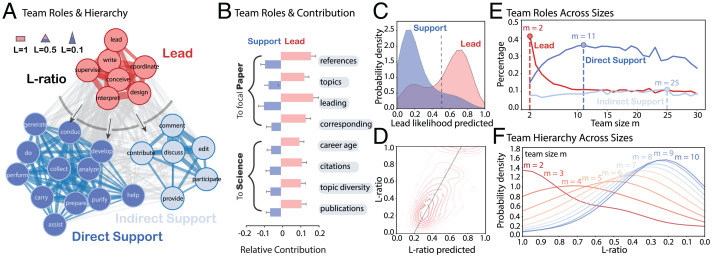
The hidden hierarchy of scientific teams. (*A*) The cooccurrence of research activities within individual authors across 89,575 contribution statements. Three clusters including “Lead” (red), “Direct Support” (blue), and “Indirect Support” (light blue) are identified. Arrows imply the direction of influence. (*B*) We verify L ratio by demonstrating the distinct contributions of lead and support authors to specific papers and science as a whole. (*C* and *D*) Our machine learning model classifies lead and support authors (precision 0.79, recall 0.793) and predicts L ratio (Pearson correlation coefficient 0.66). (*E* and *F*) The composition of team roles (*E*) and the distribution of L ratio (*F*) changes with team size.

Drawing on 89,575 self-reports of team member research activity underlying scientific papers published in PNAS, *Nature*, *Science*, and *PLOS ONE* from 2003 to 2020, we cluster the 25 most common research activities as a function of their cocontribution by authoring scientists. These activities cluster into broad roles of 1) leadership through the direction and presentation of research and 2) direct or indirect research support through data collection, analysis, and discussion (Fig. 1*A*). Specifically, leadership involves the following activities: “conceive,” “design,” “lead,” “supervise,” “coordinate,” “interpret,” and “write.” Direct and indirect support coherently separate into their own clusters. Direct support involves the following activities: “help,” “assist,” “prepare,” “develop,” “collect,” “generate,” “purify,” “carry,” “do,” “perform,” “conduct,” and “analyze.” Indirect support activities occur before the research begins and after it is complete, including “participate,” “provide,” “contribute,” “comment,” “discuss,” and “edit.” The cynical observer might reduce these roles to “brain,” “muscle,” and “fat,” the essential anatomy of modern research teams. By contrast, we demonstrate that, when more members of the team are integrated into leading roles, the character of research changes and comes to influence unfolding scientific advance in strikingly different ways. The “L ratio” quantifies the hierarchy of scientific teams defined as the fraction of authors playing lead roles among all team members.

Leading authors make contributions that are measurably distinct from those playing only supporting roles. Lead authors are 10 to 20% more likely than average to introduce references, direct topics, initiate research as first author, and manage communication as corresponding author. In contrast, support authors are 5 to 10% less likely than average to contribute to these tasks (Fig. 1*B*). We find a comparable distinction between lead and support roles when analyzing scientists’ cumulative contribution to science, measured in career age, citation impact, total number of topics studied, and total number of previously published papers. These characteristics allowed us to build a machine learning model to classify authors into “lead” and “support” roles (Fig. 1*C*), with precision of 0.79 and recall of 0.793, and to robustly predict the L ratio of scientific teams (predicted and empirical L ratios correlate at 0.66) (Fig. 1*D*). Using these models, we scale our measures of team L ratios to the complete sample of 16,397,750 papers published during 1950–2015 where roles are not reported.

The composition of team roles changes with team size. The proportion of lead authors peaks in teams of size two, authors exclusively playing direct support roles summit in teams of size 11, and those only in indirect support roles reach their maximum at teams of 25 members (Fig. 1*E*). While L ratio is clearly associated with team size such that smaller teams tend to have a higher L ratio than larger teams (Fig. 1*F*), substantial variance in L ratio for teams of the same size allows us to disentangle the effect of team hierarchy from size.

**Fig. 2.**
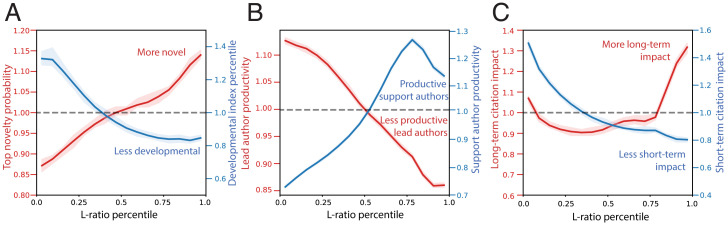
Tall vs. flat teams and the characters of research output. (*A*) Probability of writing a top 10% novel paper (red) increases with L ratio, whereas the percentile of development index (blue) decreases with it. (*B*) Lead authors are less productive in teams with a higher L ratio (red), whereas support authors experience productivity gains (blue). (*C*) Scientific publications from high-L-ratio teams receive more long-term citations after 20 y (red) but fewer short-term citations within 10 y (blue). Bootstrapped 95% CIs are shown as the shaded envelope for all curves.

Hierarchy is deeply related to characteristics of the resulting research and its recognition by others in science. The probability of writing a novel paper (top 10% atypicality) increases with the team’s L ratio, while the likelihood that a team will be recognized by others as having incrementally developed rather than radically disrupted prior ideas, measured by the development index [the inverse of disruption score (13)], decreases with it (Fig. 2*A*). We also find that lead authors are more productive in hierarchical teams with a lower L ratio, but support authors experience greater productivity on flatter teams (Fig. 2*B*). Scientific publications from low-L-ratio teams receive more short-term citations, while those from high-L-ratio teams experience greater influence over the long term (Fig. 2*C*). We separately perform author and field fixed-effects regressions controlling for team size, grant number and size, and career age among team members, finding that L ratio plays a consistent, significant, and substantial role in predicting all outcome variables (see *SI Appendix* for details). This is important, as changing the size or altering the age structure of a team involves hiring or firing members, but our findings suggest the possibility that the same goal may be achieved by reorganizing tasks: Junior scientists can be extended leading roles to maximize innovative potential.

## Discussion

Tall teams provide obvious benefits for scientists who lead them, but do not necessarily maximize the productivity and innovation potential of those who support. They enable greater lead author productivity, maximize immediate citations, and so hedge against the lead author’s risk of not receiving academic credit (14). Under some circumstances, hierarchies may represent the most efficient allocation of effort for their research purposes, but they impose costs on supporting scientists who do not play leadership roles, produce fewer papers, and accumulate less credit (see *SI Appendix* for details). The causality of these patterns remains unclear, and team structures vary by grant size and field, but our fixed-effect models suggest that, as the same researchers in the same fields with the same support shift from teams with lower to higher L ratios, their opportunities for leadership and productivity expand, corroborating prior research on the distribution of member risk associated with team hierarchy (6, 8).

Building on prior research about team member specialization (9, 10), we uncover the hierarchy of research roles and compare the lasting contributions of tall versus flat teams. Tall teams produce less novel, more developmental, and shorter-lived contributions to science. This suggests that scaling innovative teams to increase their innovation poses a paradox, especially as sponsored science pushes from “little” to “big” in the name of accelerating advance. Team hierarchy has markedly increased over the past half century, with L ratios below 0.5 rising from 50% in 1950 to 70% in 2015, but has increased even more dramatically in sponsored science (see *SI Appendix* for details). Concerns over scientific stagnation have arisen from apparent diminishing returns to scientific investment, inferred from accelerated growth in publications but slowed expansion in new ideas (15). Here we reveal the place of team hierarchy in the landscape of innovation, and provide insight for funding agencies about the critical role flat teams play in advancing supporting scientists to grow the next-generation scientific workforce for sustainable, long-term scientific advance.

## Materials and Methods

### Datasets.

We link and analyze two datasets, research articles from Microsoft Academic Graph (MAG) and author contribution statements. Our MAG data include 16,397,750 journal articles written by two or more authors during 1950–2015 (16). Each article has 7.4 topic keywords and 30.5 references, on average. Our author contribution data cover a substantial body of all available contribution statements from the time they were required by four journals, including 13 y of PNAS (18,354 during 2003–2015), 15 y of *Nature* (9,364 during 2006–2020), 3 y of *Science* (1,176 during 2018–2020), and 9 y of *PLOS ONE* (60,681 during 2006–2014). We analyze the cooccurrence between the top 25 research activities within authors, which covers 94.6% of all activities at the individual level (Fig. 1*A*). The “L ratio,” the fraction of authors in the team who participated in lead activities, is ∼0.4 (0.45 for PNAS, 0.38 for *Nature*, 0.38 for *Science*, and 0.43 for *PLOS ONE*).

### Predicting Team Roles and L Ratio.

We apply the Louvain method and identify three clusters from cooccurring research activities. We analyze distinct characteristics between these roles and build a neural network to predict author roles in 16,397,750 papers without explicit author contribution, with a precision of 0.79 and a recall of 0.793. We extend this model by including team size and the unevenness of contributions to successfully predict the L ratio with a Pearson correlation between predicted and empirical values equaling 0.66.

### Quantifying Team Performance.

We compute novelty, development index, productivity, and citations to quantify research outcomes. We measure novelty as the extent to which a paper links topics rarely appearing together, calculated as the distance between keywords in an embedding space (17). This highly correlates (ρ≈0.75) with Uzzi et al.’s (12) atypicality.

### Evaluating the Impact of L Ratio.

We perform fixed-effect regressions to control for confounders including team size and career age difference. See *SI Appendix* for the details regarding all methods.

## Supplementary Material

Supplementary File

## Data Availability

Previously published data were used for this work (16). The self-report contribution data can accessed in Zenodo, https://doi.org/10.5281/zenodo.6569339 (18).
